# Structural Studies of β-Diketones and Their Implications on Biological Effects

**DOI:** 10.3390/ph14111189

**Published:** 2021-11-20

**Authors:** Poul Erik Hansen

**Affiliations:** Department of Science and Environment, Roskilde University, Universitetsvej 1, DK-4000 Roskilde, Denmark; poulerik@ruc.dk

**Keywords:** tautomerism, biological effects, structure determinations, DFT calculations, SAR

## Abstract

The paper briefly summarizes methods to determine the structure of β-diketones with emphasis on NMR methods. Density functional calculations are also briefly treated. Emphasis is on the tautomeric equilibria of β-diketones in relation to biological effects. Relevant physical parameters such as acidity and solubility are treated. A series of biologically active molecules are treated with respect to structure (tautomerism). Characteristic molecules or groups of molecules are usnic acids, tetramic and tetronic acids, *o*-hydroxydibenzoylmethanes, curcumines, lupulones, and hyperforines.

## 1. Introduction

**Figure 1:**
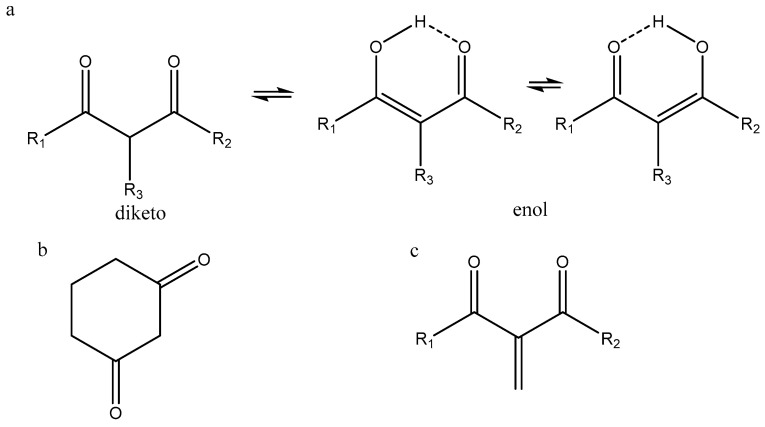
Classification of β-diketones. (**a**) lbdk type of β-diketones, (**b**) cbdk type, and (**c**) dbdk type.

β-Diketones can roughly be divided into three different groups, as shown in Figure 1. The three types are referred to as linear β-diketones (lbdk), cyclic β-diketones (cbdk), and double-bond β-diketones (dbdk). In addition, triketones are also included, as the diketone motif is present. Derivatives not containing the diketo part are not considered, as this will be too extensive and is also the case for metal complexes.

Linear β-diketones (lbdk) usually occur as a mixture of an enol-form and a diketo-form (Figure 1a), the former dominating. The enol-form shows two tautomers in fast exchange. The dominant tautomeric form is important for the biological activity. However, in this case, the barrier to interconversion between the enol and the keto-form is very important. In many papers, the different forms are not mentioned correctly or even not taken into account, which is critical when discussing biological effects. Important parameters in the discussion of biological effects are the ability of humans to take up the compounds as well as the excretion rate. Both parameters are closely related to structure.

β-Diketones can be obtained from natural sources such as plants, fungi, or bacteria, but can also be synthesized. The synthesis of the simple β-diketones is usually not complicated [1,2]. A number of reviews have discussed tautomerism and biological effects [3,4,5,6,7,8].

**Figure 2:**
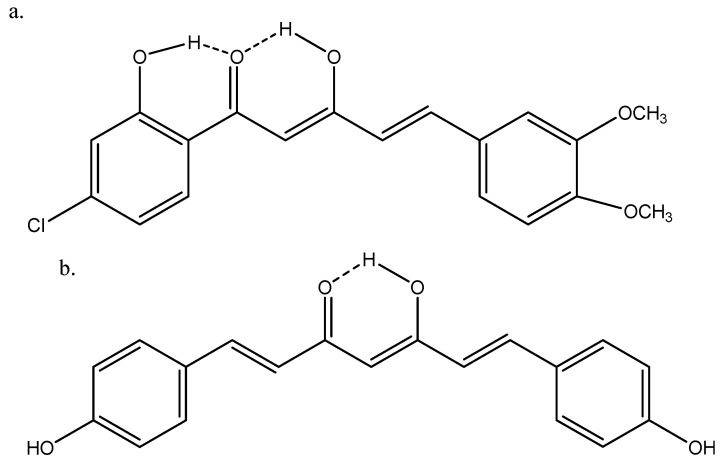
(**a**) (2*Z*,4*E*)-1-(4-Chloro-2-hydroxyphenyl)-5-(3,4-dimethoxyphenyl)-3-hydroxypenta-2,4-dien-1-one; (**b**) didesmethoxycurcumin.

Arshad et al. [9] have published an extensive review on immunosuppressive effects of natural a,b-unsaturated carbonyl-based compounds, including β-diketones. Dibenzoylmethane, 3,3′,5′-trimethoxydibenzoylmethane, curcumin, didesmethoxycurcumin (bis-demethoxycurcumin), and the ethyl equivalent of curcumin (all given as the diketoform) were found to have strong immunosuppressive effects on granylocytes. Didesmethoxycurcumin and (2Z,4E)-1-(4-chloro-2-hydroxyphenyl)-5-(3,4-dimethoxyphenyl)-3-hydroxypenta-2,4-dien-1-one (Figure 2) were found to have strong immunosuppressive effects on monocytes and macrophages.

## 2. Structure Determination

### 2.1. NMR

Structure determination and the presence of tautomerism is very important in relation to biological effects. NMR is an indispensable tool. For a review covering early results, see [10]. Using NMR in the case of β-diketones, one should remember that the equilibrium between the diketo- and the enol-forms (Figure 1) is slow on the NMR scale, and therefore both forms can be observed, irrespective of the solvent, whereas the equilibrium between the two enol-forms is so fast that only an average NMR spectrum is observed. Both the use of chemical shifts and isotope effects on chemical shifts have been used. In the latter case, deuteration of the OH proton (chelate proton) is frequently used [11], but also ^18^O isotope effects have in a few cases proven useful [12]. For low-barrier hydrogen bonds, a characteristic feature is a very high frequency ^1^H chemical shift of the chelate proton, ≈15 ppm. However, this resonance can be absent if other OH groups are present, as seen in curcumins. A simple indicator of the presence of the diketo-fom is a ^1^H resonance with a chemical shift of ≈5.3 ppm, integrating 2H for the central CH_2_ group (see Table 1).

Besides ^1^H chemical shifts, ^17^O chemical shifts can also be used. In this case, one takes advantage of the large chemical shift difference between single-bonded and double-bonded oxygen. The use has been reviewed, but some disagreements about ranges exist [13,14]. Studies have also been performed in the solid state [15]. The hydrogen bond in curcumin (see Section 9) was studied by ^17^O NMR in the solid state, in combination with DFT plane wave calculations [16].

**Figure 3:**
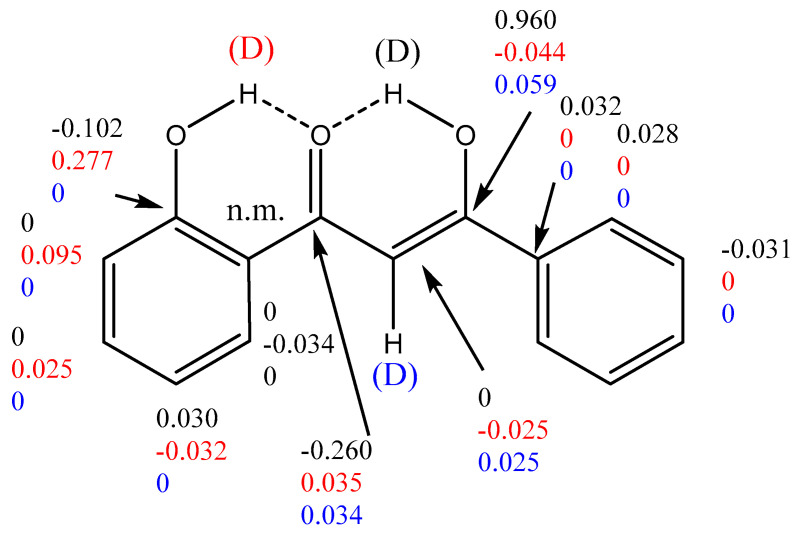
Deuterium isotope effects on ^13^C chemical shifts. They are defined as ^n^Δ = δCx(H) − δCx(D). n is the number of bonds between the label (deuterium) and the nucleus in question, Cx. The isotope effects given are color-coded to refer to the deuterium causing the effect. Data were taken from [11] with permission from Elsevier.

To determine the ratio between the two enolic forms, partial deuteration of the enol proton and measurement of deuterium isotope effects on ^13^C chemical shifts is a very useful tool. An example is seen in Figure 3 [11]. We see three different kind of isotope effects. The lower set of numbers is caused by deuteration at C-2. The fact that C-2 is deuterated is direct evidence that the keto-form and the enol-form are in equilibrium. The middle set of isotope effects is caused by deuteration at OH-2´. This effect is an indicator of hydrogen bond strength. These two types of isotope effects are the so-called intrinsic isotope effects. The top set of data are due to deuteration at the chelate OH group. The intrinsic isotope effect of this strong hydrogen bond is close to 0.5 ppm. However, the deuteration leads to a shift in the chemical equilibrium and therefore also to an equilibrium isotope effect [11]. Using the graph of [17], the mole fraction of the two enol-forms can now be determined.

**Figure 4:**
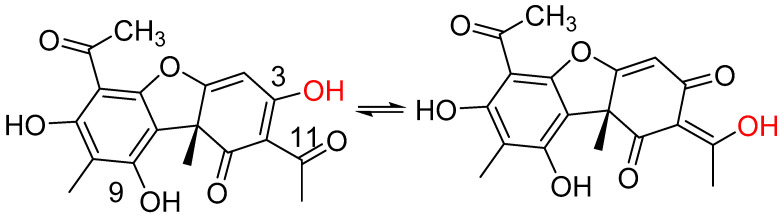
Enolic forms of usnic acid. Enolic proton marked with red.

Coupling constants involving the chelated proton may also be used. An example is the use of ^2^J(C,OH) in usnic acid (Figure 4). 

The relevant two-bond couplings are ^2^J(C-11,OH) Hz = 3.5 Hz and ^2^J(C-3,OH) = 3.6 H, showing that the equilibrium is close to 50:50 [18]. This is also confirmed by the corresponding deuterium isotope effects on chemical shifts [17]. This technique is sensitive enough to monitor a change in the equilibrium caused by acylation of the OH group at position 9. In this case, the couplings are ^2^J(C-11,OH) = 3.7 Hz and ^2^J(C-3,OH) = 3.2 Hz.

### 2.2. IR 

Sloop et al. [19] have investigated β-diketones with R_1_=CF_3_ and R_2_ = alkyl, aryl, heteroaromatic, and cyclic (Figure 1), and given a set of characteristic wavenumbers in order to identify the various tautomers. The diketo-form is 1687–1790 cm^−1^ with slight variations. The enol-form C=OCF_3_ is 1580–1640 cm^−1^, with the exception of cyclic compounds at 1550–1640 cm^−1^. For the other enol-form C=O-R, it is 1650–1700 cm^−1^. Benassi et al. [20] calculated frequencies for all four rotamers of curcumin, finding a good fit to the IR spectrum. In a similar way, the IR spectrum of glycosylated curcumin was analyzed, providing a good fit between experimental and calculated values using the B3LYP/6-311G(d) functional and basis set. The populations of the various rotamers were estimated on the basis of calculated ΔG values [21].

### 2.3. UV–VIS 

Sloop et al. [19] provided ranges for UV–VIS absorptions of the diketo-form and of the two enol-forms of β-diketones in which R_1_ (Figure 1) is CF_3_. The spectrum of the diketo- and the enol-forms often overlap. The deconvolution can be achieved using chemometric methods. For a general description of the use of chemometric methods to analyze tautomeric systems based on UV-VIS spectra, see [22]. Mondal et al. [23] investigated the UV–VIS spectrum of curcumin in several solvents and solvent mixtures. In non-polar solvents, the absorbance is at 410–420 nm, whereas in water, it is at 427 nm. For the effect of water, see Section 3. 

## 3. Ratios between Enol- and Diketo-Forms

The ratio between the diketo- and enol-forms varies with the substituents, R_1_, R_2_, and R_3_ (see Figure 1), but also with the polarity of the solvent. From Table 1, it is seen that the enol content increases as the size of R_1_ and R_2_ increases, whereas the diketo-form increases with the size of R_3_.

From Table 2, it is seen that with R_1_ and R_2_ substituents being aromatic or heteroaromatic, the enol content is close to 100%. A series of dibenzoylmethanes with substituent at the aromatic ring such as COOR or alkyl showed again a very high percentage of enol-form. The variation was from 93.1 to 99.9%. Furthermore, the variation in four different solvents such as DMSO-d_6_, acetone-d_6_, CDCl_3_, and benzene-d_6_ was moderate [19]. Sloop et al. [19] investigated β-diketones with R_2_=CF_3_. For R_3_ being very bulky or fluorine, the equilibrium is fully on the keto-form. For those molecules primarily on the enol-form, extended conjugation would favor the a-form. However, if substituents in *o*-position of the aromatic ring prevented coplanarity, this would favor the b-form and thus would intramolecular hydrogen bonding with substituents at R_1_. Finally, electron withdrawing substituents at the aromatic ring would also favor the b-form. Sloop et al. [19] claim that UV and IR are the preferred methods and that they are better than NMR, but this is only because they have used inadequate NMR methods. Had they used deuterium substitution, this would not be the case (see Section 2). With R_1_ and R_2_ equal to substituted double bonds as found in curcumins, the pattern is similar [25]. The influence of R_3_ is pronounced.

**Figure 5:**
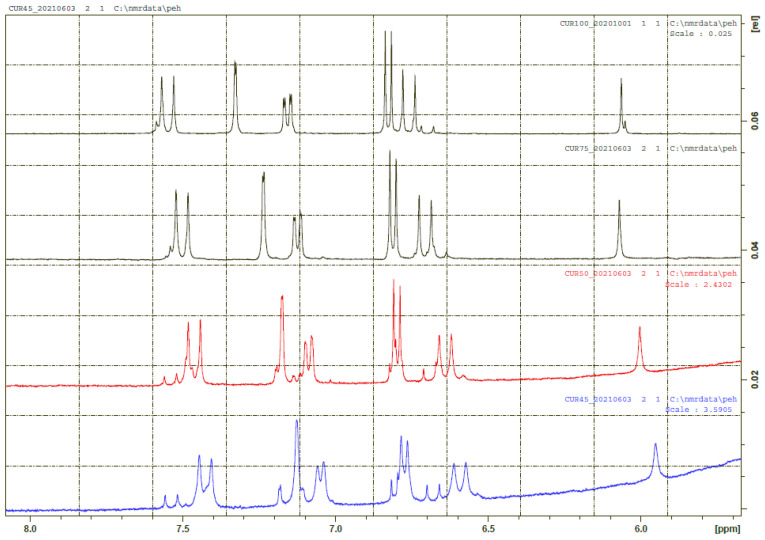
^1^H NMR spectra of curcumin in a mixture of H_2_O and DMSO-d_6_. From top, 0%, 25%, 50%, 55% H_2_O. The major form is the enol-form. The diketo-form is seen as the minor resonances.

Bunting et al. have determined the equilibrium constant between the keto and the form in aqueous solution with the ionic strength of 0.1 at 25 °C, as seen in Table 3. It is obvious that the keto-form in most cases is dominant, contrary to organic solvents. However, the factors influencing the equilibrium constants seem to be the same [29]. The keto–enol equilibrium of curcumin in ethanol/water mixtures was investigated using UV–VIS spectroscopy. The amount of diketo-form is increasing with the increase in the water content [30]. The same trend is found in DMSO–water mixtures (see Figure 5).

For avobenzone 1-(4-(tert-butyl)phenyl)-3-(4-methoxyphenyl)propane-1,3-dione, R_1_ = 4-terbutylphenyl, and R_2_ = 4-methoxyphenyl, as shown in Figure 1, and the amount of diketo-form can be increased in chloroform from 4 to 10% if the central carbon is dideuterated [31]. 

## 4. Interconversion between Tautomers

Interconversion between the two enol-forms is very fast due to the very low barrier. It has never been possible to observe the two enol-forms separately by NMR, but they can be measured by femtosecond absorption in the ultraviolet range [32]. However, the interconversion between the enol and the keto-form is slow on the NMR time scale. The barrier for interconversion for acetylacetone has been calculated as 62 Kcal, but with water present, it was ≈30 Kcal [33]. Conradie et al. [26] determined the first-order rate constant for conversion of the enol to keto-form for R_1_ = R_2_ = 2-thienyl as 3.1 × 10^−5^ s^−1^ and for R_1_ = 2-thienyl and R_2_ = phenyl as 2.6 × 10^−6^ s^−1^. Katritzky et al. [3] discussed general rules for observing the two tautomers. Obviously, if the rate is slow compared to the biological time scale, both tautomers should be considered separately. However, if it is fast, it does not really matter.

## 5. pKa Values

Bunting et al. have determined pKa values for a large series of β-diketones for both the keto- and the enol-forms and the equilibrium constant in aqueous solution with ionic strength of 0.1 at 25 °C. The pKa values for the two forms are not very different (see Table 3) [29]. Furthermore, only with pyridyl substituents are the pKa values below the physiological pH of 7.4. In this case, a substantial amount of the anion can be expected in the blood. For enols at the anion form, a different conformation with the two oxygens “trans” can be expected. For triketones, the pKa values are even lower, and an example is usnic acid with a pKa value of 4.4 [34] In pegylated usnic acid, the pKa in a mixture of H_2_O and DMSO was determined as 4.3 [5]. 

## 6. Inclusion Complexes

**Figure 6:**
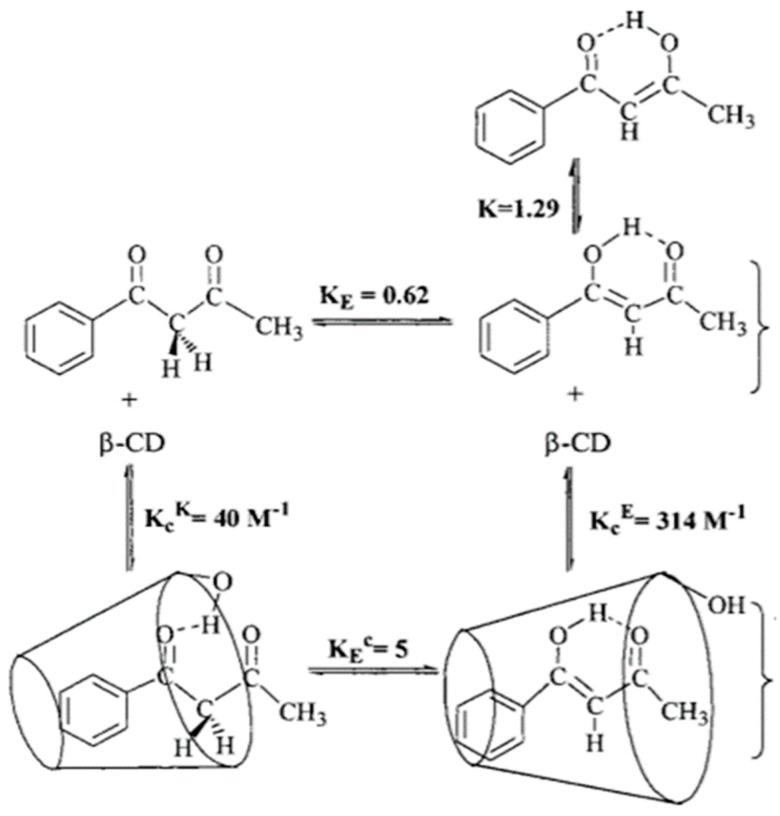
Benzoylacetone complexation with β-cyclodextrin. E refers to enol- and K to diketo-form. Taken from [35] with permission from the American Chemical Society.

A study related to drug delivery is the study of benzoylacetone enclosed into β-cyclodextrin. Iglesias et al. [35] found that the planar enol-form protruded deeper into the cyclodextrin cavity, and this way leads to more enol-form (see Figure 6). For the less planar diketo-form, only the phenyl ring is buried. Strangely enough, the diketo-form is drawn with the two oxygens on the same side. A hydrogen bond from the rim to the keto group is also suggested.

Inclusion of curcumin has been used to a large extent. Angelova and Antonov have studied the inclusion in calixarenes both experimentally [36] and theoretically [37]. In the latter case, they found that both tautomeric forms can enter and leave the host cavity without sterical problems and that the diketo-form was favored.

## 7. Density Functional Calculations

Density functional calculations (DFT) are very useful in the study of compounds in solution. DFT calculations can be performed at many levels and provide a variety of details about structure, NMR, IR, UV parameters, and energy, etc. In the present author’s opinion, a “cheap” solution, referring to memory requirements and computational time, such as B3LYP/6-31G(d) [38], can often provide useful results in cases of NMR and IR calculations. An example is the calculation of the often elusive OH stretching frequencies of β-diketones [39,40], but also NMR parameters such as chemical shifts, isotope effects on chemical shifts, and coupling constants can be calculated in a satisfactory way for β-diketones [41]. A higher basis set was used for curcumin derivatives such as 6-311++G(2d,p) [42]. To calculate UV–VIS spectra, Benassi et al. used B3LYP/6-311G(d,p) [20], whereas Puglisi et al. used M06-2X/def2-TZVP functional and basis set [43].

**Figure 7:**
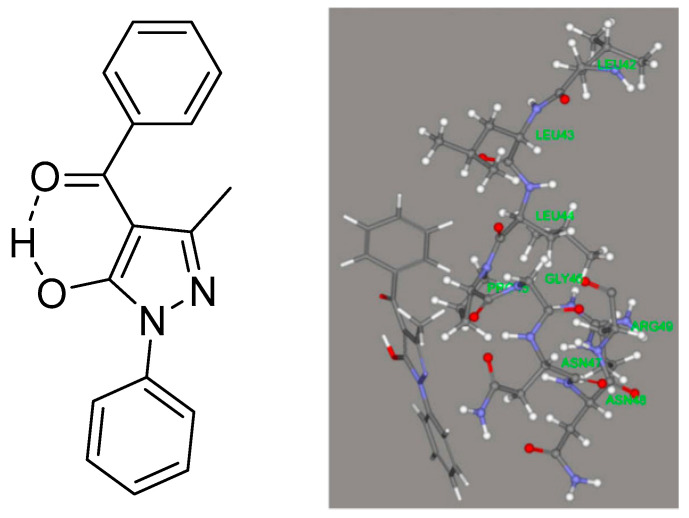

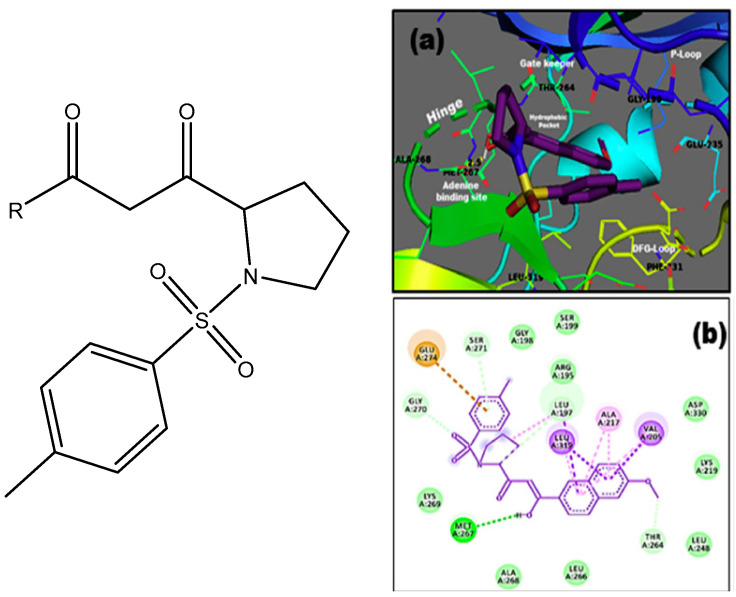
**Top:** One enol-form of HQPh and the preferred tautomer docked to the ICAM-1 protein from [44]. With permission from Elsevier. **Bottom**: β-Diketones derived from L-proline. R can be phenyl, p-toluene, p-Cl, Br, I-phenyl, 2,4-difluoro- and 2,4-dichorophenyl, 2-fluoro-4-bromo- and 2-methyl-4-iodophenyl, and 2-furanyl and 2-naphthyl-6-methoxy. Docking of the latter in the binding site of PTK6. (**a**). Showing the docking. (**b**). Showing the amino acid involved. Taken from [46] with permission from Elsevier.

Caruso et al. [44] compared X-ray data and DFT data for 4-benzoyl-3-methyl-1-phenylpyrazol-5-one (see Figure 7) and found especially for the diketo-form an excellent agreement between structures determined by X-ray and calculated structures. The effect of water was taken into account using the COSMO algorithm [45]. DFT calculations were used to discuss the structure of 4-furancarbonyl, 4-t-butylcarbonyl, 4-(3-cyclopentylpropanoyl), and 4-tert-butylacetyl of 3-methyl-1-phenylpyraxol-5-ones.

## 8. Docking Studies

**Figure 8:**
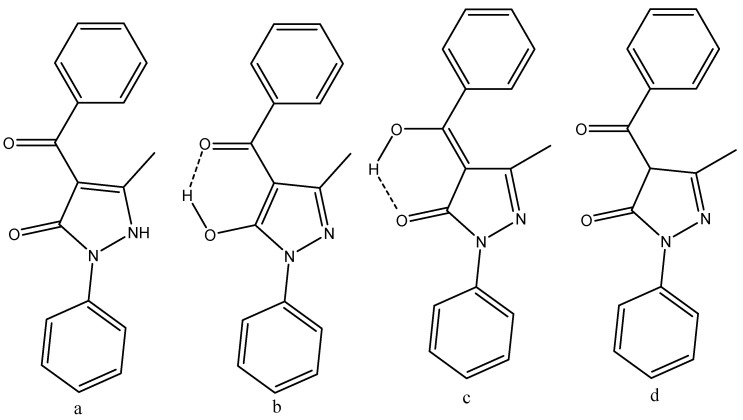
Tautomeric forms, (**a**,**d**) are ditketo forms, (**b**,**c**) are enol forms of 4-benzoyl-3-methyl-1-phenylpyrazol-5-one (HQPh). From [44].

Caruso et al. [44] docked HQPh to the ICAM-1 protein and found that the keto–enol-form acted better than the diketo-form. For the docked structure (see Figure 7, top), the obtained inhibition was 75%. The highest binding energy for the four tautomers shown in Figure 8 is that of d. However, the one docked is that of b (Figure 7, top), as that of d is not present to any large extent in water. Porchezhiyan et al. [46] synthesized a large series of L-proline-based β-diketones (Figure 7, bottom). They found that the derivative with R = 6-methoxynaphthalene showed anti-cancer activity. Docking of the compounds shown in Figure 7 towards COX-1 and COX-2 was performed using the protein crystal structures of PDB ID: 1HT5 in order to evaluate the anti-inflammatory activity. Dhoke et al. [47] developed docking procedures for, among other diketones, 2,4-pentanedione and 3,5-heptanedione. These were docked as the diketo-form. As shown in Table 1, they exist primarily as the keto–enol-form. Zusso et al. [48] docked curcumin, bis-demethoxycurcumin (Figure 2), and a cyclized pyrazole analogue (the β-diketone unit has been reacted with hydrazine). All three could bind into the LPS binding site of myeloid differentiation protein-2. Important interactions were hydrogen binding with Arg-90, Glu-92, and Tyr-102. However, only the two curcumins inhibited LPS-induced TLR4 dimerization, activation of NF-κB, and secretion of pro-inflammatory cytokines in primary microglia.

## 9. Structures

A general feature of β-diketones of type A (see Figure 1) is the apparent resemblance of the diketo-form and the enol-forms. However, this is only due to the traditional way of drawing. The diketo-form is more on a form with the two keto groups trans to each other.

**Figure 9:**
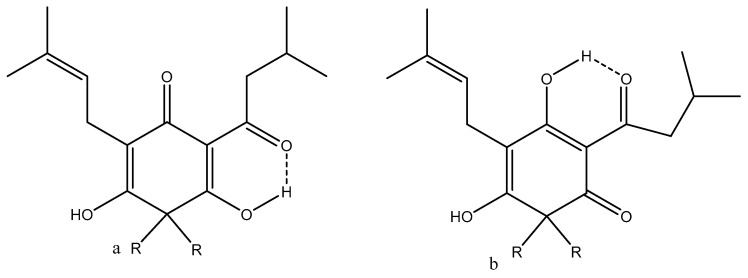
Lupulone, R is equal to isoprene. (**a**,**b**) are different positional enol isomers.

In the following section, a number of structures are discussed in relation to their biological effects. Lupulone from hops soft resin showed two different enolic forms depending on solvent, as seen in Figure 9.

**Figure 10:**
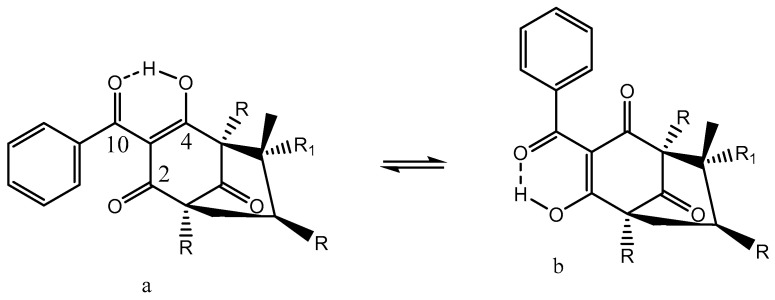
Tautomeric equilibrium of garciniaphenone (R = isoprenyl and R_1_ = methyl) and 7-*epi*-clusianone (R = isoprenyl, R_1_ = H). (**a**,**b**) are different positional enol isomers.

In cyclohexane, a 30:70 mixture of a/b exists, whereas in DMSO-d_6_, the compound is fully on form b. (-)-(R)-humulone (one R is an OH group, which is pointing inwards) and lupulone inhibit cell growth and induce caspase-dependent apoptosis [49]. A similar finding was uncovered for garciniaphenone and for 7-*epi*-clusianone (Figure 10). Both compounds show strong intramolecular hydrogen bonds. Both forms a and b could be observed in the ^1^H NMR spectrum (OH chemical shifts, 17.90 and 17.35 ppm). This means that the tautomeric exchange is rather slow. The two tautomers exist in a ratio of 5:1 for a/b [50]. The structure was also confirmed by an X-ray study [51]. A similar behavior was found for guttiferones A-E and clusianone [52,53]. Guttiferone A, a similar compound, shows HIV-inhibitory properties [54]. Guttiferone A, garciniaphenone, and 7-epiclusianone are discussed together with many other natural products as cathepsin inhibitors [55,56].

Caruso et al. [44] synthesized a large series of 3-methyl-1-phenylpyraxol-5-ones. The benzoyl derivative is shown in Figure 8. Other substituents were 4-furancarbonyl, 4-t-butylcarbonyl, 4-(3-cyclopentylpropanoyl), and 4-tert-butylacetyl. They were tested on the intercellular adhesion molecule-1 (ICAM-1). The best inhibition was obtained for the molecule in Figure 7. DFT calculations were performed, and c was found to have the lowest energy (Figure 8). However, both a and b are within 5 Kcal. The fact that c had the lowest energy is somewhat unusual, as it encloses an exocyclic double bond. Unfortunately, no interconversion barriers are known. HQPh is more active than its metal complexes.

**Figure 11:**
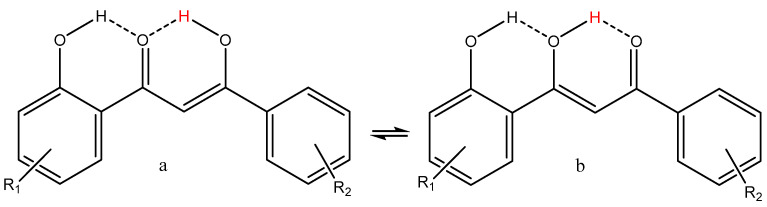
1-(2-Hydroxyphenyl)-3-phenyl-1,3-propanedione (*o*-hydroxydibenzoylmethane). For an example of the diketo-form, see Figure 1. (**a**,**b**) are enolforms. The enolic proton is in red.

An interesting structure that seems to cause diverse biological effects is shown in Figure 11. 

This structural element is part of tetracyclines, and in this case, the equilibrium is shifted towards the a-form [8]. Furthermore, it has shown promising effects in comparison with mycobacteria [57]. 1-(2-Hydroxyphenyl)-3-phenyl-1,3-propanedione was shown to induce apoptosis in colorectal carcinoma COLO 205 cells. It is better than dibenzoylmethane and also better than the derivative of *o*-hydroxydibenzoylmethane with a methyl group in 5-position (R_1_ is CH_3_ in Figure 9). The mechanism is a complicated chain reaction starting with coordinative modulation of Cyclin D3, Bcl-X_L_ and Bax, release of cyctochrome c, and sequential activation of caspases [58]. Furthermore, the derivative with R_1_ = H and R_2_ = *o*-OH shown in Figure 9 inhibited TPA-induced skin tumor promotion significantly [59]. However, there was no discussion about the tautomer being responsible for the action, and only a resemblance to aspirin is mentioned. *o*-Hydroxydibenzoylmethane is selectively cytotoxic against breast cancer MCF-7 cells [60].

**Figure 12:**
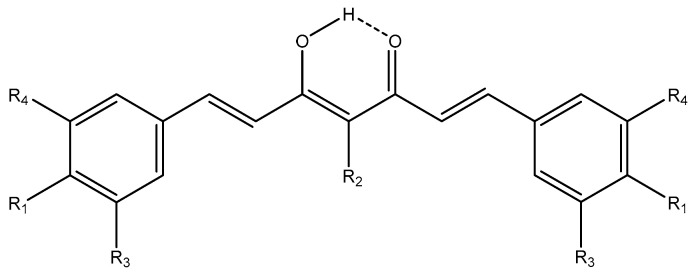
Analogues of curcumin and isocurcumin.

**Figure 13:**
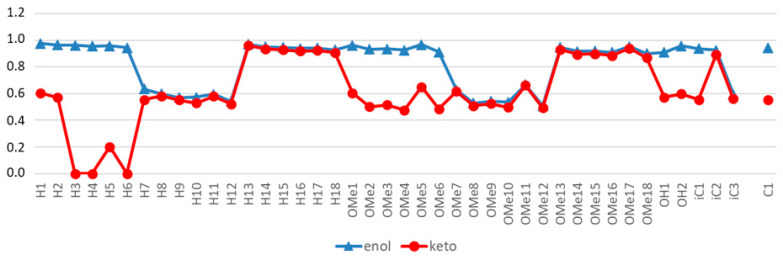
Ranking of the 23 enol- (blue triangles) and 23 diketo (red diamonds)-forms according to their calculated prostate cancer treatment (PCT) ability. H1,H2 etc. refer to Table 4. For comparison, the values for the enol-form (eC1) and the diketo-form (kC1) of curcumin is included. Taken from [42].

Many recent reviews deal with curcumin [61,62,63,64]. Another recent review found that taking curcumin would increase the expression of anti-metastatic proteins [65]. Curcumin belongs to the Pan-Assay Interference Structures (PAINS) family [66]. In the present paper, only very recent papers are discussed specifically. Important features of curcumin are the existence of both diketo-form and enol-forms. A recent study [42] investigated a large series of curcumin analogues (Figure 12). Probabilities to act as antineoplastic, prostate cancer treatment, and anticarcinogenic agents was studied theoretically by applying a selection of quantitative structure–activity relationship and absorption, distribution, metabolism, and excretion (ADME) approaches. For the compounds, the enol-form is generally the more effective. They are good against prostate cancer (see Figure 13). With regard to substituents, the more OH and OCH_3_ groups, the better.

The degradation of curcumin leads to a number of products, some of which could be important for biological action. However, only glucosidation maintains the β-diketone structure [67,68,69]. In terms of action, it has been found that the presence of piperidine from, e.g., black pepper increases the effect, but this is an indirect effect as piperidine decreases the degradation rate [70].

**Figure 14:**
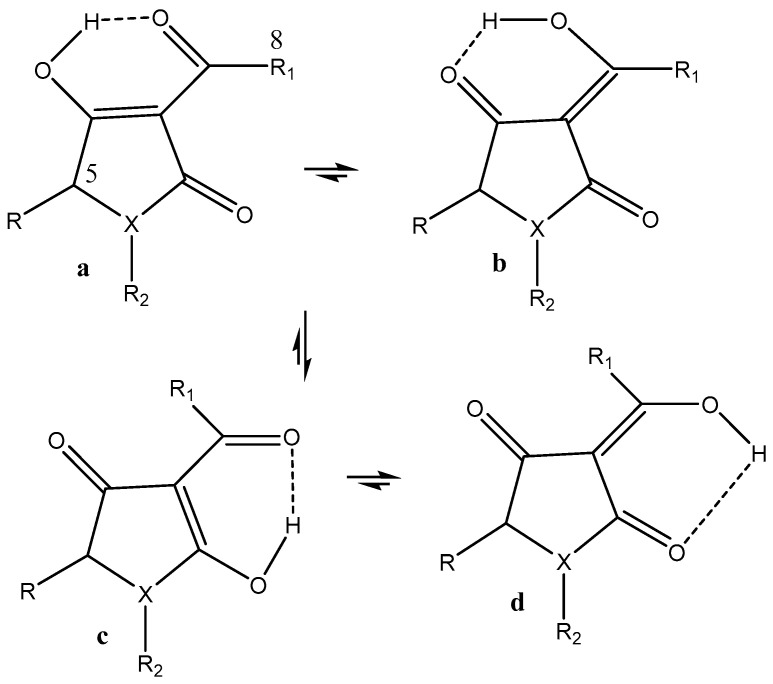
Tautomeric equilibria of 3-acyl tetronic (X=O) and tetramic (X=N) acids. (**a**–**d**) are the different enolic forms.

Tetronic acids, tetramic acids, and 3-acylderivatives are found frequently in nature. An early review was given by Schobert and Schlenk [71]. The 3-acyl derivatives are triketones. Two-sets of tautomeric equilibria exist, as seen in Figure 14. An efficient way of analyzing such systems is the use of deuterium isotope effects [6,72]. A recent example is the isolation of penicillenol A_1_ and A_2_ (R=C(OH)CH_3_ and R_1_ = 1-methylhexyl and R_2_ = CH_3_) from the sponge *P. fusca* Thiele [73] and also from a deep see fungus, *Aspergillus restrictus*. The penicillenols are active against Candida albicans biofilm formation. A structure–bioactivity relationship study suggested that the saturation of hydrocarbon chain at C-8, R-configuration of C-5, and trans-configuration of the double bond between C-5 and C-6 of the pyrrolidine-2,4-dione unit were important for their anti-biofilm activities [74].

**Figure 15:**
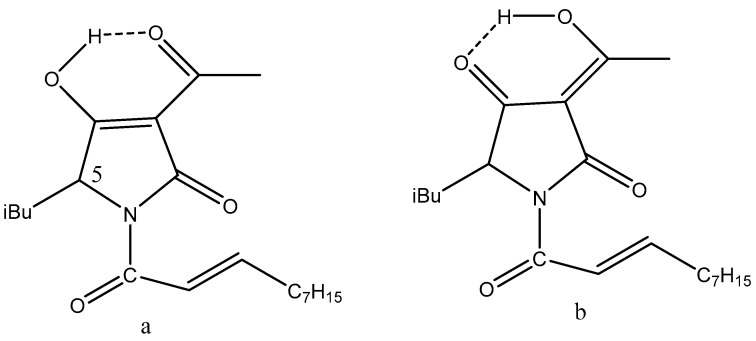
Reutericyclin. (**a**,**b**) are two different enolic forms.

A recent paper discussed synthesis based on catalysis. Interesting examples are reutericyclin (Figure 14) and other examples given in [75]. Again, reutericyclin is depicted as the a-form (Figure 15), whereas it is the b-form that is active. A SAR study showed that lipophilic analogs were best against Gram-positive bacteria [76].

Tetramic acid is also combined with ampicillin to form a hybrid in order to improve the effect against Gram-negative bacteria [77].

**Figure 16:**
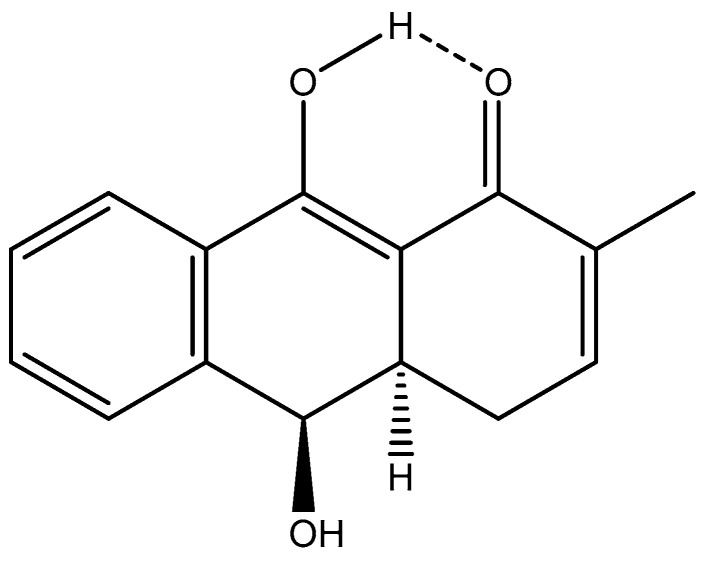
Structure of *Rubia philippinensis* as suggested in [78].

The dihydroanthracen-1(4*H*) one (Figure 16) isolated from *Rubia philippinensis* is an inhibitor of soluble epoxide hydrolase (sEH). The structure was given as a single tautomer [68] but has been suggested to exist as a tautomeric equilibrium [8].

**Figure 17:**
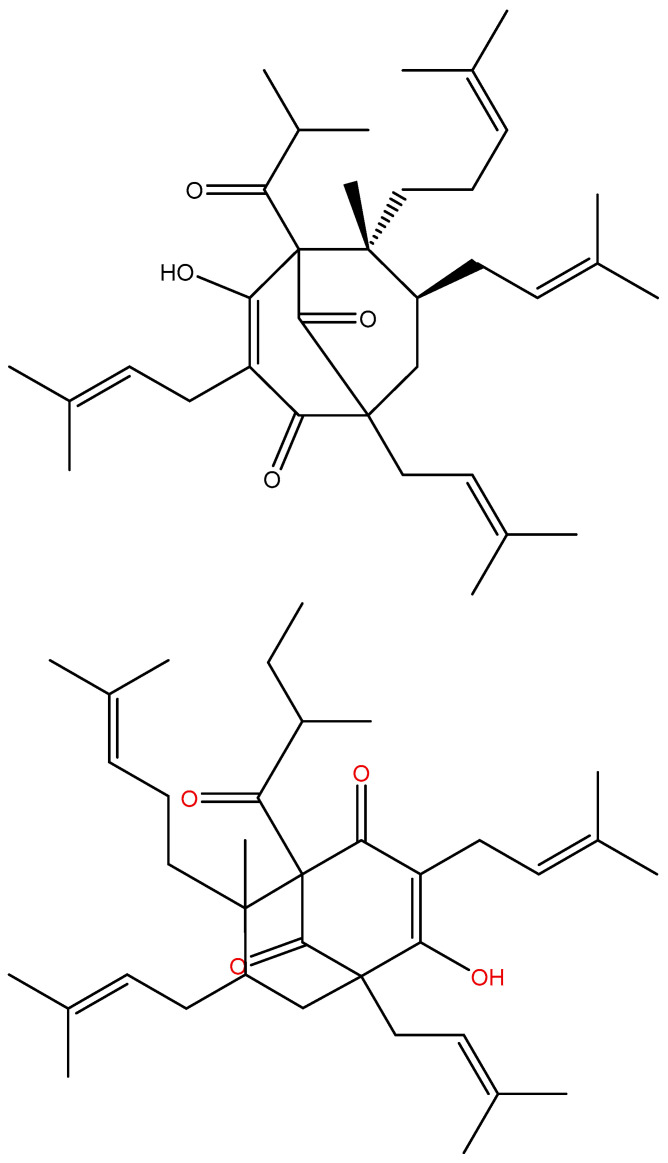
Hyperforin (**top**) and adhyperforin (**bottom**).

From St. John´s wort (*Hypericum perforatum*), a number of active compounds have been isolated. One of these being hyperforin (Figure 17), which has antidepressant properties [79], as well as adhyperforin [80]. Simple prenylated compounds were also synthesized and show activity. 

Two β-diketones have attracted a large amount of attention over the years, namely, usnic acid (see Figure 4) and tetracyclines. A large number of usnic acid derivatives have been developed [81,82]. However, some of these are derivatized at the tri-ketone site. Usnic acid is not very water-soluble [83]. Attempts have been made to increase this. One way to is to make a polyamide complex. The polymer/drug complexes possess a greater antimicrobial activity against *Staphylococcus epidermidis* than usnic acid itself [84]. Another way is the Rop/Raft strategy [85]. Solubilization is also increased by the use of micellar solutions of N,N’-didecyl-N,N,N’,N’-tetramethylethane-1,2-diyldiammonium dibromide and decyl 2-[decyl(dimethyl)ammonio]ethylphosphate [86] or by used of graphene–usnic acid conjugate microspheres. The latter showed antibacterial activity against *Staphylococcus aureus* [87]. A simple approach is to attach a polyglycol to one OH group [5]. The use of a salt to increase solubility has also been suggested [88]. Considering the low pKa value of usnic acid [34], one would image that this would happen automatically. This leads to a discussion of the predominant form at physiological pH [5].

## 10. Conclusions

β-Diketones are characterized by an equilibrium between a keto form and an enol form (Figure 1). The interconversion between these two forms is usually slow, which means that from a biological point of view, the two forms may have widely different biological properties, especially as the conformations of the keto-form and the enol-form are different. The enol-forms are characterized by a very strong intramolecular hydrogen bond, keeping the structure with the two oxygens at the same side. In contrast, the keto-form will typically have the two C=O bonds in a “trans” orientation. The fact that the enol-form has a strong intramolecular hydrogen bond is usually not taken into account in programs predicting biological properties. Docking studies demonstrate the importance of a correct tautomer. The enol-form usually dominates in solvent with a low dielectric constant. The ratio of the enol- and the keto-forms can be regulated by substitution at the central carbon. Large substituents will favor the keto-form. In water, the equilibrium between keto- and enol-forms is shifted strongly towards the keto-form. The acidity of the OH proton is for most of the β-diketones not acidic enough to lead to an anionic structure. The β-diketones clearly have many biological functions, but the motif seems to be part of many different molecules with a variety of biological functions.

## Figures and Tables

**Table 1:** ^1^H chemical shifts of CH_2_, CH, and OH protons and enol percentage. From [24] with permission from the American Chemical Society.

Compound/Chemical Shifts ^a^ and % Enol	Diketo	Enol	OH	% Enol
2,4-Pentanedione	3.58	5.50	15.34	79
2,4-Hexanedione	3.18 ^b^	5.08 ^b^	13.46 ^b^	81
5-Methyl-3,5-hexanedione	3.57	5.50	14.92	80
2,2-Dimethyl-3,5-hexanedione	3.56	5.60	15.58	94
3,5-Heptanedione	3.66	5.66	15.04	76
3,5-Heptanedione	3.18 ^b^	5.12 ^b^	14.30 ^b^	76
2-Methyl-3,5-heptanedione	3.57	5.50	14.92	88
2,2-Dimethyl-3,5-heptanedione	3.56	5.58	15.88	92
2,6-Dimetyl-3,5-heptanedione	3.60	5.50	15.50	94
2,2,6-Trimethyl-3,5-heptanedione	3.54 ^b^	5.28 ^b^	15.52 ^b^	96
2,2,6,6-Tetramethyl-3,5-heptanedione	3.74	5.86	^c^	^c^

^a^ Chemical shifts in ppm from internal TMS. ^b^ Chemical shifts in ppm from external TMS. ^c^ No data given.

**Table 2:** β-Diketones with aromatic and heteroaromatic substituents. Solvent CDCl_3_. Percentage of enol-form ^a^.

R_1_ ^b^	R_2_	% Enol	Reference
CH_3_	2-Thiophene	84, 94.4	[26,27]
C_3_F_7_	2-Thiophene	100	[28]
2-Thiophene	2-Thiophene	82	[26,27]
Ph	2-Thiophene	94.3, 92.8	[27]
CF_3_	2-Thiophene	94.4	[26]
Ph	2-Furane	95.5	[27]

^a^ See also Table 3 and the paper by Sloop et al. above [19]. ^b^ R_1_ and R_2_ refer to Figure 1.

**Table 3:** pKa values and equilibrium constants for enol to keto-forms. Data from [29] with permission from Canadian Science Publication.

R_1_ ^a^	R_2_	pK_a_e ^b^	pK_a_k	K_e_ ^c^
CH_3_	CH_3_	8.03	8.71	0.21
Ph	Ph	8.64	≈7.9	≈6
3-Py	3-Py	7.04	6.78	1.8
4-Py	4-Py	5.45	≈4.65	≈6
CH_3_	Ph	8.39	8.53	0.72
CH_3_	3-Py	7.15	7.47	0.48
CH_3_	4-Py	7.16	7.00	1.4
Ph	3-Py	7.37	7.26	1.3
Ph	4-Py	7.27	-	3

^a^ R_1_ and R_2_ refer to Figure 1. ^b^ e and k refer to enol- and diketo-forms. ^c^ K_e_ is defined as K_e_ = [eH]/[kH].

**Table 4:** Substitution patterns for the curcumin analogues.

	R_1_ ^a^	R_2_	R_3_	R_4_		R_1_ ^a^	R_2_	R_3_	R_4_
H1	H	H	H	H	OH1	OH	H	H	H
H2	Br	H	H	H	OMe1	H	H	OMe	OMe
H3	F	H	H	H	OMe2	Br	H	OMe	OMe
H4	Cl	H	H	H	OMe3	F	H	OMe	OMe
H5	OMe	H	H	H	OMe4	Cl	H	OMe	OMe
H6	N(Me)2	H	H	H	OMe5	OMe	H	OMe	OMe
H7	H	Cl	H	H	OMe6	N(Me)2	H	OMe	OMe
H8	Br	Cl	H	H	OMe7	H	Cl	OMe	OMe
H9	F	Cl	H	H	OMe8	Br	Cl	OMe	OMe
H10	Cl	Cl	H	H	OMe9	F	Cl	OMe	OMe
H11	OMe	Cl	H	H	OMe10	Cl	Cl	OMe	OMe
H12	N(Me)2	Cl	H	H	OMe11	OMe	Cl	OMe	OMe
H13	H	Me	H	H	OMe12	N(Me)2	Cl	OMe	OMe
H14	Br	Me	H	H	OMe13	H	Me	OMe	OMe
H15	F	Me	H	H	OMe14	Br	Me	OMe	OMe
H16	Cl	Me	H	H	OMe15	F	Me	OMe	OMe
H17	OMe	Me	H	H	OMe16	Cl	Me	OMe	OMe
H18	N(Me)2	Me	H	H	OMe17	OMe	Me	OMe	OMe
iC1	OMe	H	OH	H	OMe18	N(Me)2	Me	OMe	OMe
iC2	OMe	Cl	OH	H	OH2	OH	H	OMe	OMe
iC	Ome	Me	OH	H	C1	OH	H	OMe	H

^a^. R´s refer to Figure 12.
